# Optical coherence tomography findings after longitudinal ablation for an underexpanded stent in a heavily calcified lesion: a case report

**DOI:** 10.1186/s12872-016-0419-8

**Published:** 2016-11-29

**Authors:** Masahiro Koide, Keiji Inoue, Akiko Matsuo, Hiroshi Fujita

**Affiliations:** Department of Cardiovascular Medicine, Japanese Red Cross Society Kyoto Daini Hospital, 355-5 Haruobicho, Kamigyoku Kyoto, 602-8026 Japan

**Keywords:** Coronary angiography, Optical coherence tomography, Percutaneous coronary intervention, Rotational atherectomy, Case report

## Abstract

**Background:**

Heavy coronary artery calcification is responsible for stent underexpansion, which is associated with increased in-stent restenosis. Here we report a case in which optical coherence tomography (OCT) demonstrated that the metal component of an underexpanded stent previously implanted in a heavy calcified lesion had been completely removed after ablation with rotational atherectomy.

**Case presentation:**

An 83-year-old man with exertional angina was referred to our hospital. Coronary angiography revealed severe stenosis in the proximal portion of the right coronary artery and left circumflex artery and chronic total occlusion (CTO) in the mid portion of the left anterior descending artery (LAD). We performed complete revascularization with percutaneous coronary intervention. Because the CTO lesion in LAD contained napkin-ring heavy calcifications, rotational atherectomy with a 1.75-mm burr was undergone, followed by the deployment of drug-eluting stents and postdilation with a high-pressure balloon. However, expansion of the stent was incomplete. To address the recurrence of in-stent restenosis and resistance to the dilation with the high-pressure balloon, we decided to simultaneously ablate both the heavy calcification and underexpanded stent. Longitudinal stent ablation with 1.75- and 2.0-mm burrs was successful, and OCT demonstrated that the metallic component of the underexpanded stent had been completely removed.

**Conclusion:**

If a stent fails to completely extend in heavy calcification, longitudinal stent ablation by rotational atherectomy could be an effective remedy.

**Electronic supplementary material:**

The online version of this article (doi:10.1186/s12872-016-0419-8) contains supplementary material, which is available to authorized users.

## Background

Severe calcification in a coronary atherosclerotic plaque is responsible for stent underexpansion, which is associated with an increased risk for in-stent restenosis (ISR) and target lesion revascularization [1, 2]. Stent underexpansion is also an important issue underlined not only in metallic stent, but also in bioresorbable vascular scaffold [3]. To avoid stent underexpansion during percutaneous coronary intervention (PCI) to a heavily calcified lesion, plaque modification using a high-pressure balloon, cutting balloon, and rotational atherectomy prior to stent implantation is very important [4–6]. If a serious stent underexpansion had occurred, high-pressure balloon dilation may fail to achieve full expansion because of severe calcifications. Few previous reports have demonstrated rotational atherectomy debulking heavy calcifications together with the metallic component of underexpanded stents, thereby allowing complete expansion of lesions [7, 8]. On the other hand, optical coherence tomography (OCT) has demonstrated to be the gold standard to evaluate coronary in the stent expansion [9]. However, there are no reports regarding OCT, which proved that the metallic component of stent was removed by ablating with rotational atherectomy. Here we report OCT findings regarding the removal of an underexpanded stent in a severely calcified lesion by ablating with rotational atherectomy.

## Case presentation

**Fig. 1:**
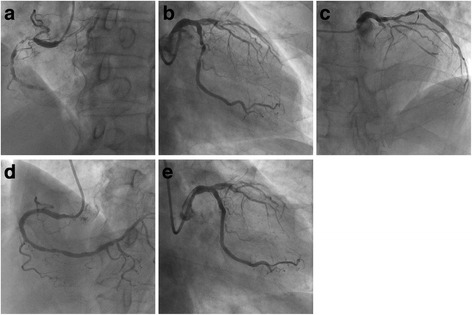
Initial coronary angiography and percutaneous coronary intervention to the right coronary and left circumflex arteries. Initial coronary angiography (**a**-**c**). Percutaneous coronary intervention to the right coronary artery (**d**) and left circumflex artery (**e**)

An 83-year-old man with exertional angina for 6 months was referred to our hospital. Coronary angiography revealed a three-vessel disease, which included severe stenosis from the proximal to the mid portion of the right coronary artery (RCA), a chronic total occlusion (CTO) in the mid portion of the left anterior descending artery (LAD), and diffuse stenosis from the proximal to the mid portion of the left circumflex artery (LCX) (Fig. 1a–c). The conus branch provided collaterals to LAD, and the atrial circumflex branch provided collaterals to the distal RCA. We decided to perform PCI because the patient refused coronary artery bypass grafting. First, we performed PCI on RCA and LCX, which was divided into two procedures (Fig. 1d, e).

**Fig. 2:**
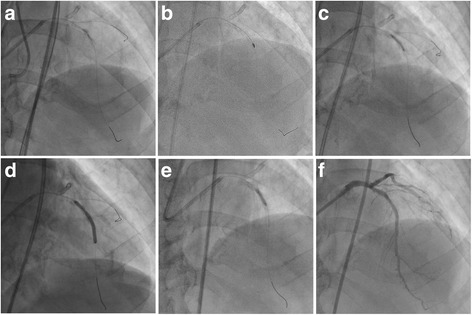
Percutaneous coronary intervention to the chronic total occlusion in the left anterior descending artery. Predilation with both 2.0-mm balloons (**a**). Rotational atherectomy with a 1.75-mm RotaLink™ (**b**). Dilation with a 2.25-mm high-pressure balloon (**c**), followed by deployment of Promus Premier™ stents (**d**). Additional postdilation with a 2.5-mm non-compliant balloon at 22 atm (**e**). The final angiography (**f**)

After 1 month, we performed PCI to address CTO in LAD (Fig. 2). After the Gaia 2nd guidewire successfully passed CTO, we attempted predilation at 22 atm with both a 2.0-and 2.25-mm balloon (Fig. 2a); however, the lesion could not be completely expanded. Therefore, we performed rotational atherectomy using the 1.75-mm RotaLink™ (Boston Scientific) (Fig. 2b). The slow flow phenomenon was observed after the burr passed across the lesion. We concerned about the risk for the burr sizing up; thus, we performed additional dilation using a 2.25-mm high-pressure balloon (Fig. 2c) followed by the deployment of two Promus Premier™ stents (Fig. 2d). Additional postdilation with a 2.5-mm non-compliant balloon at 22 atm was applied to the underexpanded stent in the mid portion of LAD (Fig. 2e); however, this failed to achieve complete expansion of the stent (Fig. 2f).

The follow-up coronary angiogram after 6 months revealed ISR within the underexpanded stent. We performed balloon angioplasty using a 2.5-mm high-pressure balloon, however, it was not possible to sufficiently expand the stent.

**Fig. 3:**
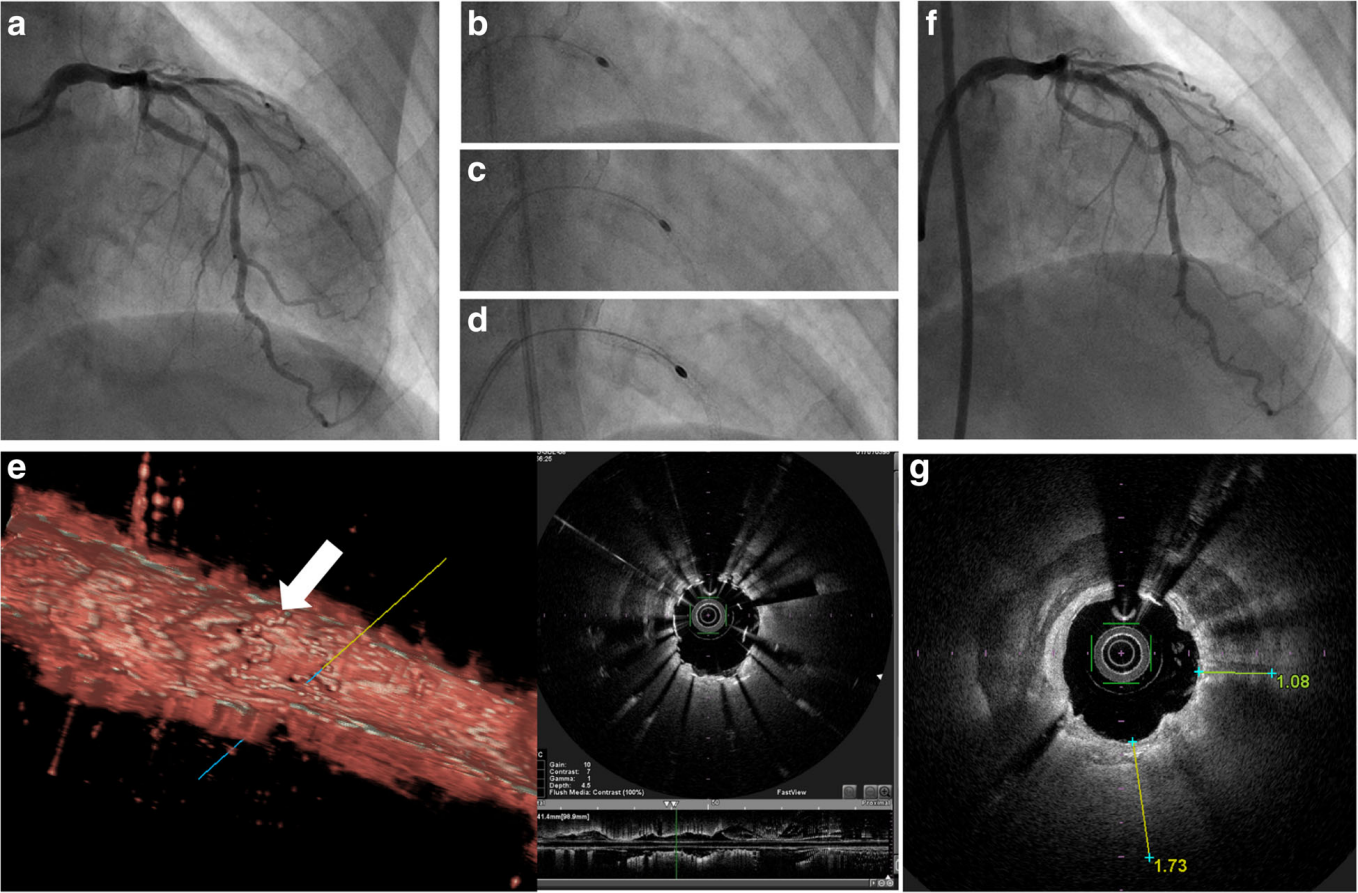
Longitudinal stent ablation using rotational atherectomy. The follow-up coronary angiogram revealed in-stent Rees to notice within the underexpanded stent (**a**). Longitudinal stent ablation using rotational atherectomy (**b**, 2.0-mm burr; **c**, 1.75-mm burr; **d**, 2.0-mm burr). Optical coherence tomography revealed that the stent turned inward (**e**). The final angiography (**f**). Calcium thickness at the site where the burr passed (**g**)

**Fig. 4:**
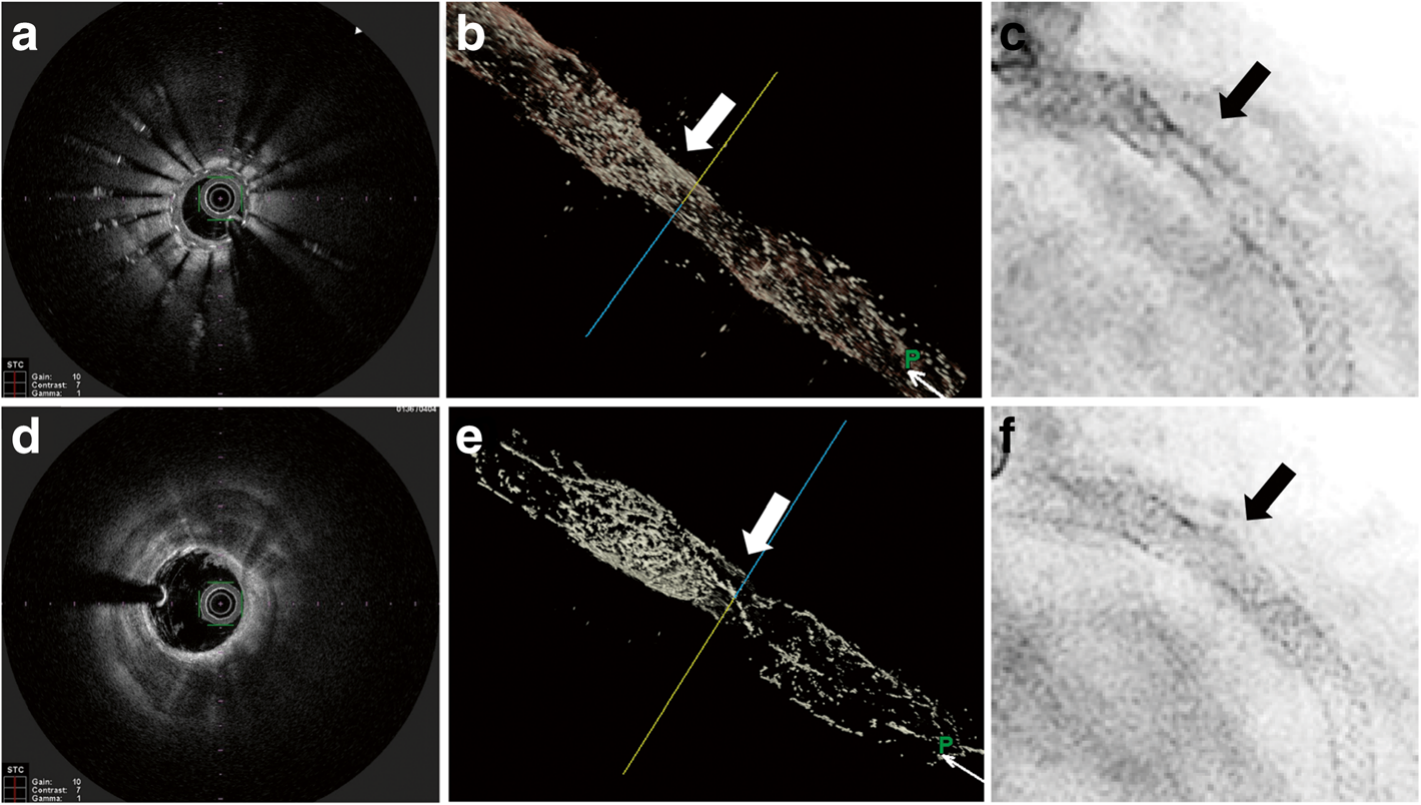
Optical coherence tomography and fluoroscopy demonstrated the complete removal of the metallic component of the underexpanded stent. Before (**a**-**c**) and immediately after percutaneous coronary intervention (**d**-**f**)

After 5 months, the follow-up coronary angiogram revealed ISR (Fig. 3a); this involved coronary ischemia, indicated by an FFR of 0.64. Thus, we performed PCI on the refractory ISR. Because the dilation of the lesion was considered impossible, we decided to simultaneously ablate both the stent and the heavily calcified lesion using rotational atherectomy. An 8-Fr sheath was inserted thorough the femoral artery, and the left coronary artery was engaged with an 8-Fr JR 4.0 guide catheter (Boston Scientific). A SION blue™ wire was advanced into the distal LAD. OCT (FastView™, Terumo Corporation) revealed circumferential heavy calcification around the underexpanded stent. Although a 2.0-mm RotaLink™ was gradually advanced to the lesion at 150,000 rpm (Fig. 3b), the burr became entrapped within the underexpanded stent, and we ended up pulling out the drive shaft of the Rotablator™. We continued to ablate with the burr that was reduced in size from 2.0 to 1.75 mm, and the rotating speed was increased from 150,000 to 200,000 rpm (Fig. 3c). The rotating speed of the 1.75-mm burr unexpectedly decreased at the small proximal portion of the underexpanded stent. After several ablations, the 1.75-mm burr finally passed through the lesion. We changed to a new 2.0-mm burr and continued to ablate the lesion (Fig. 3d). Although this 2.0-mm burr again became entrapped within the underexpanded stent, it was easily removed by pulling out the drive shaft of the Rotablator™. After the burr passed through the lesion, fluoroscopy and OCT demonstrated that the metallic component of the underexpanded stent had completely disappeared (Fig. 4) (see Additional file 1). Furthermore, OCT revealed that the stent turned inward (Fig. 3e). Finding it difficult to use an additional burr because of its cost, we dilated the lesion using a 3.0-mm cutting balloon™ (Boston Scientific) at 24 atm and a 2.5-mm Hiryu™ balloon (Terumo) at 30 atm; however, this did not enable the heavily calcified lesion to completely expand. We then additionally dilated the lesion using a 3.0-mm drug-coated balloon. Final angiography showed an acceptable result (Fig. 3f), and FFR had improved to 0.81, indicating the release of the myocardial ischemia.

## Conclusions

The greatest concern with longitudinal stent ablation is to slip through the underexpanded stent without ablating the metal and leaving the burr immovable [10]. Hence, we advanced the burr more gradually and carefully than usual. The slow flow phenomenon due to the metallic debris and stent thrombosis because of heat generation were also concerns, but in this case, these complications did not arise. Furthermore, some risk of future stent fracture owing to the removal of a part of the stent is a matter to be taken into account.

A previous study reported that electron microscopic analysis observed the significant loss of a diamond tip from the surface of RotaLink™ after ablating the metal component of a stent [11], suggesting that the ablation power decreases after an initial contact with the first burr. In this study, after the first 2.0-mm burr was entrapped and a second burr of 1.75 mm had passed through the lesion, using a new 2.0-mm burr resulted in successfully passing through the lesion.

Kubo et al. assessed the thickness of calcium fractured by balloon dilation during PCI, showing a maximal calcium fracture thickness of 770 μm [5]. Thus, if a 2.25-mm burr had also been used in this case, complete expansion in the lesion would not have been expected because the calcium to the side of where the burr was passing was extraordinarily thick (approximately 1100–1700 μm) (Fig. 3g).

After removing the 2.0-mm entrapped burr, the rotating speed of the 1.75-mm burr remarkably decreased at the proximal portion of the underexpanded stent. OCT examination revealed the stent turned inward in that portion, indicating that the 1.75-mm burr was ablating the deformed stent. Thus, if a marked reduction in rotating speed is observed at the proximal portion of an underexpanded stent during longitudinal stent ablation, balloon dilation should be used to adequately reform the stent strut.

We demonstrated that stent ablation with rotational atherectomy could be safely performed for an underexpanded stent in a heavily calcified lesion and that OCT revealed the complete removal of the stent strut. Stents should not be deployed in lesions in which it is impossible for a balloon to be completely expanded. However, if this situation unexpectedly occurs, longitudinal stent ablation using rotational atherectomy could be an effective remedy.

## Additional file

Additional file 1: The OCT movie after longitudinal ablation for an underexpanded stent using rotational atherectomy. (MP4 10040 kb)

## Abbreviations

CTO: Chronic total occlusion
FFR: Fractional flow reserve
ISR: In-stent restenosis
LAD: Left anterior descending artery
LCX: Left circumflex artery
OCT: Optical coherence tomography
PCI: Percutaneous coronary intervention
RCA: Right coronary artery

## References

[1] Madhavan MV, Tarigopula M, Mintz GS, Maehara A, Stone GW, Genereux P (2014). Coronary artery calcification: pathogenesis and prognostic implications. J Am Coll Cardiol.

[2] Moussa I, Ellis SG, Jones M (2005). Impact of coronary culprit lesion calcium in patients undergoing paclitaxel-eluting stent implantation (a TAXUS-IV sub study). Am J Cardiol.

[3] Imori Y, D’Ascenzo F, Gori T, Munzel T, Ugo F, Campo G (2016). Impact of postdilation on performance of bioresorbable vascular scaffolds in patients with acute coronary syndrome compared with everolimus-eluting stents: A propensity score-matched analysis from a multicenter “real-world” registry. Cardiol J.

[4] Matthew I, Annapoorna S, Samin K (2014). Current status of rotational atherectomy. J Am Coll Cardiol Cariov Interv.

[5] Kubo T, Shimamura K, Ino Y, Yamaguchi T, Matsuo Y, Shiono Y (2015). Superficial calcium fracture after PCI as assessed by OCT. J Am Coll Cardiol Cardiovasc Imaging.

[6] Iannaccone M, Barbero U, D’Ascenzo F, Latib A, Pennacchi M, Rossi ML, et al. Rotational atherectomy in very long lesions: Results for the ROTATE registry. Catheter Cardiovasc Interv. 2016;doi:10.1002/ccd.26548. 10.1002/ccd.26548 27083771

[7] Kobayashi Y, Teirstein P, Linnemeier T, Stone G, Leon M, Moses J (2001). Rotational atherectomy (stentablation) in a lesion with stent underexpansion due to heavily calcified plaque. Catheter Cardiovasc Interv.

[8] Medina A, Lezo JS, Melian F, Hernandez E, Pan M, Romero M (2003). Successful stent ablation with rotational atherectomy. Catheter Cardiovasc Interv.

[9] Iannaccone M, D’Ascenzo F, Templin C, Omede P, Montefusco A, Guagliumi G, et al. Optical coherence tomography evaluation of intermediate-term healing of different stent types: systemic review and meta-analysis. Eur Heart J Cardiovasc Imaging. 2016. 10.1093/ehjci/jew070 27099274

[10] Feldman T (2001). Rotational ablation of stent metal components: the intersection between coronary intervention and auto body repair. Cathter Cardiovasc Interv.

[11] Ho PC, Weatherby TM, Dunlap M (2010). Burr erosion in rotational ablation of metallic coronary stent: an electron microscopic study. J Interven Cariol.

